# Prevalence of Antibiotic Resistance Genes in Differently Processed Smoothies and Fresh Produce from Austria

**DOI:** 10.3390/foods14010011

**Published:** 2024-12-25

**Authors:** Sonia Galazka, Valerie Vigl, Melanie Kuffner, Irina Dielacher, Kathrin Spettel, Richard Kriz, Norbert Kreuzinger, Julia Vierheilig, Markus Woegerbauer

**Affiliations:** 1 Division of Data, Statistics and Risk Assessment, Austrian Agency for Health and Food Safety AGES, 1220 Vienna, Austria; sonia-ewa.galazka@ages.at (S.G.);; 2 Institute of Water Quality and Resource Management, TU Wien, 1040 Vienna, Austria; 3 Division of Clinical Microbiology, Department of Laboratory Medicine, Medical University of Vienna, 1090 Vienna, Austria; 4 Section Biomedical Science, Health Sciences, FH Campus Wien University of Applied Sciences, 1100 Vienna, Austria; 5 Division of Infectious Diseases and Tropical Medicine, Department of Medicine I, Medical University of Vienna, 1090 Vienna, Austria; 6 Interuniversity Cooperation Centre Water & Health, Vienna, Austria

**Keywords:** antibiotic resistance genes, antimicrobial resistance, smoothie, fresh produce, AMR, ARGs, 16S rRNA gene sequencing, bacterial community composition, food/feed chain

## Abstract

Plant-derived foods are potential vehicles for microbial antibiotic resistance genes (ARGs), which can be transferred to the human microbiome if consumed raw or minimally processed. The aim of this study was to determine the prevalence and the amount of clinically relevant ARGs and mobile genetic elements (MGEs) in differently processed smoothies (freshly prepared, cold-pressed, pasteurized and high-pressure processed) and fresh produce samples (organically and conventionally cultivated) to assess potential health hazards associated with their consumption. The MGE *ISPps* and the class 1 integron-integrase gene *intI1* were detected by probe-based qPCR in concentrations up to 10^4^ copies/mL in all smoothies, lettuce, carrots and a single tomato sample. The highest total (2.2 × 10^5^ copies/mL) and the most diverse ARG and MGE loads (16/26 targets) were observed in freshly prepared and the lowest prevalences (5/26) and concentrations (4.1 × 10^3^ copies/mL) in high-pressure-processed (HPP) smoothies. *Bla_CTX-M-1-15_* (1.2 × 10^5^ c/mL) and *strB* (6.3 × 10^4^ c/mL) were the most abundant, and *qacEΔ1* (95%), *bla_TEM1_* (85%), *ermB* and *sul1* (75%, each) were the most prevalent ARGs. *QnrS*, *vanA*, *sat-4*, *bla_KPC_*, *bla_NDM-1_* and *bla_OXA-10_* were never detected. HPP treatment reduced the microbial loads by ca. 5 logs, also destroying extracellular DNA potentially encoding ARGs that could otherwise be transferred by bacterial transformation. The bacterial microbiome, potential pathogens, bacterial ARG carriers and competent bacteria able to take up ARGs were identified by Illumina 16S rRNA gene sequencing. To reduce the risk of AMR spread from smoothies, our data endorse the application of DNA-disintegrating processing techniques such as HPP.

## 1. Introduction

Antimicrobial resistance (AMR) is a global threat to human health. This observation is underscored by an estimated global mortality rate of 4.95 million fatalities associated with AMR in 2019 [1]. However, AMR is not only a clinical but also an ecological problem, as antibiotic-resistant bacteria (ARB) and antibiotic resistance genes (ARGs) are disseminated across ecosystem borders involving human and animal as well as plant-, soil-, water- and air-borne microbiomes [2]. The top priority for tackling AMR is the reduction in AMR spread across these ecosystems, which requires a holistic “One Health”-perspective considering human, animal and environmental biospheres as closely interconnected entities [3]. In line with this approach, foodstuffs are considered vectors for the dissemination of AMR from animals and environmental compartments to humans [4].

Fresh produce is usually consumed raw or minimally processed and therefore assumed to pose a higher risk for the transmission of ARB and ARGs compared to other food categories. Upon ingestion, ARB and ARGs originating from fresh or non-processed plant-based products are translocated into the mammalian oropharynx and gastrointestinal tract where food-borne resistant bacteria may proliferate and amplify ARGs through cell division or disseminate ARGs via horizontal gene transfer (HGT) to indigenously present commensal bacteria or even pathogens [5,6]. One form of HGT is bacterial transformation, where free extracellular DNA (exDNA) can be incorporated by competent bacteria capable of taking up exogenous DNA from their environment [7,8]. This dissemination route does not require direct cell-to-cell contacts. ExDNA is prevalent in all ecosystems inhabited by bacteria and has also been reported in diverse matrices like mammalian body fluids (e.g., saliva, urine, and blood) and foodstuffs [9,10]. In situ HGT by natural transformation has been observed inter alia in drinking water, milk and cheese [9,11,12].

Although ARGs are indigenously present in all ecosystems harboring bacteria, these environments can be contaminated additionally by clinically relevant ARGs of anthropogenic origin [13]. Furthermore, ARGs can be transferred from environmental non-pathogenic bacteria to clinically critical pathogens [2,14]. This is especially of concern if ARGs are encoded on MGE-like plasmids, integrative conjugative elements or insertion-sequences but also on integrons carrying resistance gene cassettes that are embedded on transposons or in membrane vesicles [14,15].

Fresh produce and plant-based smoothies have not been in the focus as clinically relevant ARG reservoirs so far. However, due to changes in lifestyle and according to diet recommendations, their consumption continually increased over the past decades [16]. Microbial contamination of these food groups has been reported as a source of food-borne illnesses, ARGs and ARB [17]. Fresh vegetables and fruits are frequently contaminated with pathogenic strains of *Escherichia coli* and *Klebsiella pneumoniae* expressing clinically important ARGs like the β-lactamases *bla_OXA_* _(-48, -58, -66, -72, -181)_, *bla_KPC-2_*, *bla_NDM_* _(-1, -5, -9)_, the colistin resistance gene *mcr-1* and various ARGs inactivating aminoglycosides (*aac(3′)-Ia*, *aac(6′)-Ib*, *aadA1*, *aph(3′)-VI, armA*, *rmtB*, *strA, strB*, etc.), sulfonamides (*sul1*, *sul2*), tetracyclines (*tet(A)*, *tet(B)*, *tet(D)*), diaminopyrimidines (*dfrA12*, *oqxAB*), fosfomycin (*fosA3*), fluoroquinolones (*qnrS1*) and chloramphenicol (*floR*), among others [18]. These genes are usually located on mobile genetic elements that boost their dissemination via conjugation and transformation in exposed bacterial populations [15,16,17,18,19]. Coliform bacteria resistant to antibiotics were found in raw, freshly prepared or cold-pressed smoothies, which are praised as more nutritious than heat- or high-pressure treated counterparts [20,21,22].

To minimize the risk of plant-borne ARG transfers, the application of food processing technologies promoting the disintegration of DNA molecules is recommended [23]. For preservation and for prolonging shelf life, thermal processing like pasteurization or high-pressure processing (HPP) is usually favored [24].

Pasteurization relies on the application of thermal energy to inactivate enzymes and kill heat-sensitive bacteria. The applied temperatures (55–90 °C/30 s–6 min) vary according to the food matrix and the respective pH value. However, these temperatures are usually too low to induce irreversible DNA denaturation [25]. Irreversible double-strand breaks and DNA fragmentation can be achieved with temperatures above 150–200 °C [26]. Unfortunately, bacterial DNA was reported to easily withstand temperatures of up to 95 °C for 30 min and shorter fragments remain PCR-amplifiable even after heat treatment at 100 °C for up to 240 min [26,27,28].

High-pressure processing utilizes the principle of isostatically pressurizing food and beverages at room temperature. The killing efficiency of the procedure is determined by food extrinsic (e.g., target pressure, holding time, and processing temperature) and intrinsic factors (acidity, water activity, and nutrient content) as well as by strain-specific traits of the affected microorganisms [24]. After the application of a typical pressure of 400–600 MPa in a humid environment for several minutes, sudden depressurization induces deleterious changes in cell membrane permeability, morphology and cellular metabolism that kill microbes. Endonucleases are released, which leads to DNA cleavage and DNA condensation [29,30]. HPP therefore appears to be the method of choice to reduce intra- and extracellular ARG loads in smoothies and fresh produce.

The aim of this pilot study was to determine the prevalence and the amount of 24 clinically relevant ARGs inactivating 12 classes of antibiotics and two mobile genetic elements (MGEs) in differently processed, commercially available smoothies (freshly prepared, cold-pressed, pasteurized and high-pressure processed) and fresh produce samples (carrots, lettuce and tomatoes: organically and conventionally cultivated) by probe-based qPCR to assess potential health hazards associated with their consumption. The results of the fresh produce samples were used to put the smoothie results into an appropriate context for comparison. We hypothesized that distinct processing techniques would have divergent impacts on ARG and bacterial DNA concentrations resulting in lower ARG loads in treated samples that would reduce ARG exposure rates of the indigenous microbiome of the consumers. For the identification of potential pathogens, we characterized the microbial community by metagenomic 16S rRNA gene sequencing. Potential ARG-carrying taxa were identified via network correlation analyses, and bacteria naturally competent for the uptake of ARGs encoded on free extracellular DNA were determined by sequence alignments. To our knowledge, no studies are available so far investigating total ARG loads and bacterial community composition in the tested food matrices and applying ARG-carrier network correlation analyses in a comparably comprehensive way.

## 2. Materials and Methods

### 2.1. Sample Collection and Experimental Design

For assessing ARG prevalences and concentrations, the determination of the bacterial community composition, and for identifying pathogens, potential bacterial ARG carriers and bacteria naturally competent for the uptake of extracellular DNA, twenty commercially available smoothies characterized by different food processing levels were collected. Of these smoothies, 14 were freshly prepared in two Austrian supermarkets and three coffee–juice bars, 2 were cold-pressed, 2 were pasteurized and 2 were high-pressure processed. The establishments for sample acquisition were selected according to their popularity and high customer frequency. The freshly prepared smoothies to be analyzed were identified by interrogation of the shop owner or the available sales assistant for their most popular products. Origin, main plant-associated ingredients and utilized preservation methods for each of the tested smoothie samples are presented in Table 1. Of the freshly prepared and well-mixed smoothies, approximately 200 mL were decanted on-site without touching the rim into a sterile 250 mL polypropylene (PP) bottle, each (Nalgene, VWR, Vienna, Austria). The operator wore disposable latex gloves, which were changed between the samples, and an FFP2 respirator mask (YiHuB, Guangdong Province, China) to reduce contaminations potentially introduced by the operator to a minimum. The PP bottles were tightly sealed and transferred immediately into a cooling box equipped with 4–6 frozen cold packs. Smoothies offered in glass or plastic bottles from the manufacturer were directly put into the cooling box and transported with the PP bottles at 4–10 °C within 6 h–8 h to the laboratory. There, all smoothies were aliquoted in 50 mL sterile PP tubes (VWR, Vienna, Austria) under aseptic conditions in a laminar flow hood dedicated for sample preparations using sterile disposable 50 mL plastic pipettes and an electric pipettor protected by a 0.22 µm filter. Before starting the work, the pipettor and the inner working bench surfaces of the flow hood were decontaminated with 10% sodium hypochlorite followed by swipe-cleaning the surfaces with tissue soaked in 70% ethanol. The sample aliquots were stored at −20 °C in a freezer dedicated to sample collection before DNA extraction. Aliquoting was performed wearing non-powdered latex gloves decontaminated with 10% sodium hypochlorite to avoid cross-contamination of the samples with DNA.

For comparison and to put the results obtained from smoothies into an appropriate context, 12 additional raw and unprocessed produce samples (4 carrots, 4 lettuce and 4 tomatoes) were collected from the same 2 highly frequented Austrian supermarkets. Half of the samples were “organically”, and the remaining half was “conventionally” cultivated on Austrian farms. One produce sample per cultivation method was collected in each supermarket. The selected food plants were additionally characterized by different levels of exposure to soil particles (root vegetables, leafy greens, and fruit vegetables, respectively). These plant-based food samples were selected because raw, non-peeled and non-processed plant-based food bears the highest risk for AMR transfer to consumers and their microbiomes.

Smoothie and fresh produce samples were purchased in the timeframe of June to July 2021. Fresh produce was transported under the same conditions as described for the smoothie samples and was rinsed in the laboratory with sterile distilled water to remove visible residues of soil or other dirt sources. Fresh produce samples were also stored at −20 °C for a maximum of 24 h before DNA extraction. 

### 2.2. DNA Isolation

DNA isolation was performed using the DNeasy PowerFood Microbial Kit (Qiagen, Vienna, Austria) for both beverages and fresh produce.

To avoid DNA cross-contaminations, all samples were prepared in a dedicated laminar flow hood: Before handling the samples, the inside of the workbench and all reusable instruments were decontaminated with 10% sodium hypochlorite. After an incubation period of 5 min, hypochlorite residues were removed with paper towels, and the surfaces were cleaned additionally with wipes soaked in 70% ethanol (EtOH). EtOH-decontaminated nitrile gloves were used for laboratory experiments. Isolation of microbial smoothie DNA was executed according to Appendix A of the Experienced User Protocol for liquid foods [31]. Briefly, a total of 1.8 mL of the smoothie sample was centrifuged in sterile 2 mL collection tubes for 3 min at 13,000× *g*. After resuspending the microbial pellet in 450 µL of Solution MBL, the standard protocol was performed, including a bead-beating step on the FastPrep-24 (MP Biomedicals, Austria) twice at 6 m/s for 30 s.

Fresh produce was aseptically diced into small pieces and homogenized with a sterilized pestle. DNA isolation proceeded according to the DNeasy PowerFood Microbial Kit Experienced User Protocol (Appendix A) for solid foods. A total of 0.25 g of fresh produce was aseptically weighed and combined with 0.75 mL of PBS in a 2 mL collection tube. Similarly to the smoothie DNA isolation, a bead-beating step was included with identical settings. Smoothie and produce samples were extracted in biological triplicates. DNA concentration and quality were measured using the NanoDrop 2000c spectrophotometer (Thermo Fisher Scientific, Vienna, Austria) and agarose gel electrophoresis.

### 2.3. qPCR Assays

The presence and abundance of 24 antimicrobial resistance genes, the 16S rRNA gene as well as two mobile genetic element-associated genes were determined by hydrolysis probe-based real-time qPCR (see below and Table S1). Analysis was performed on the LightCycler 480 II (Roche, Vienna, Austria) using undiluted DNA eluates from smoothie and produce samples.

Inhibition testing of samples was performed to rule out inhibitory effects in DNA eluates. For this purpose, the DNA eluates were tested undiluted and diluted (1:5) in PCR-grade water via 16S rRNA gene and *sul1* (sulfonamide resistance gene) qPCR. If the crossing point (Cp)-difference between the two dilution steps was below 2.0 or above 2.5, a 1:5 dilution was applied. No qPCR-inhibition was detected allowing the application of undiluted DNA eluates for the qPCR experiments.

ARGs inactivating critically and highly important antimicrobials like aminoglycoside (*aadA*, *aph(3′)-IIa*, *aph(3′)-IIIa*, *strB*), diaminopyrimidine (*dfrA-1*), phenicol (*cmxA*), fluoroquinolone (*qnrS*), glycopeptide (*vanA*), macrolide (*ermB, ermF*), nucleoside (*sat-4*), polypeptide (*mcr-1*), sulfonamide *(sul1*), tetracycline (*tet(A)*, *tet(M)*, *tet(O)*, *tet(W*)) and ß-lactam (*bla_CTX-M-1-15_*, *bla_KPC_*, *bla_NDM-1_*, *bla_TEM-1_*, *mecA*) antibiotics were analyzed [32].

**Figure 1:**
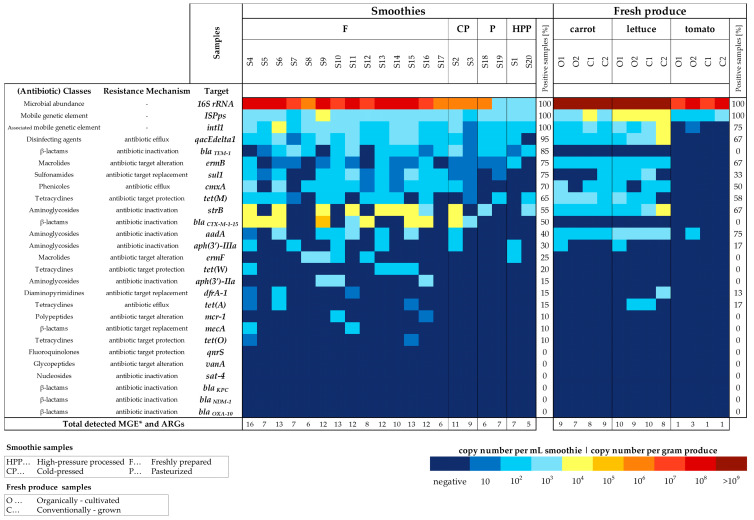
Prevalence and absolute abundance of ARGs and MGE-associated genes (MGE*) in smoothie and fresh produce samples.

The selected resistance genes covered 5 resistance mechanisms (efflux, antibiotic inactivation, target replacement, target alteration and target protection as depicted in Figure 1) [33]. Additionally, the biocide-/antiseptic resistance gene *qacEΔ1* and two genes associated with mobile genetic elements (class 1 integrase *intI1* and insertion-sequence *ISPps*) were tested.

The targets for the 27 different genes were designed—if feasible—in duplex assays, otherwise as single assays by Ingenetix (Vienna, Austria). Duplex assays are capable of simultaneously detecting two different targets in a single qPCR reaction due to two different fluorescent reporter dyes (e.g., FAM and VIC). Benefits of duplex assays include less DNA eluate consumption per sample and hands-on time, reduction in errors and lower reagent costs. However, duplex assays are not always compatible with every target due to sequence similarity and primer dimerization resulting in sub-optimal PCR efficiencies. [34]. In such a case, single assays were performed.

The single assay qPCR reaction mix consisted of 5 µL LightCycler 480 Probes Master 2x (Roche, Austria), 2.5 µL PCR-grade water, 0.5 µL TaqMan Assay (end-concentration 0.8 µM forward primer, 0.8 µM reverse primer and 0.2 µM probe (Ingenetix, Vienna, Austria), and 2 µL of undiluted DNA, yielding a total volume of 10 µL. Duplex assays consisted of the same reagents and concentrations with the exception of using 0.5 µL TaqMan assay with 2 µL PCR-grade water. The qPCR specifications are presented in Table S1 with duplex assays indicated. The cycling conditions included 1 cycle at 95°C for 10 min, followed by 45 cycles at 95 °C for 10 s, 60 °C for 30 s and 72 °C for 10 s.

Calibration curves were run on each qPCR plate using AMPure bead purified (Beckman Coulter, Vienna, Austria) and quantified (Qubit dsDNA HS Assay; Thermo Fisher Scientific, Vienna, Austria) amplicons functioning as dsDNA standards for absolute quantification and run quality estimation.

All qPCR assays were validated using a semi-logarithmic dilution series of these amplicon standards starting with 8000 copies per 2 µL down to 0.5 copies per 2 µL. The statistical evaluation is detailed in Section 2.5 and in the Supplementary Materials. qPCR procedures and validation followed the MIQE guidelines [35].

Samples were considered negative if the Cp-value was ≥ 40 or if none or only one of three technical replicates was positive with a Cp ≥ 37. If the Cp-difference between technical replicates of a sample was above 1, qPCR was repeated for that sample.

All qPCR reactions were performed in triplicates, and all runs included a non-template control (i.e., negative control: PCR-grade water in triplicates) to confirm the absence of ARG or MGE contamination in reagents. In total, 9 measurement points per sample (3 biological replicates with 3 technical replicates, respectively) were generated. A logarithmic dilution series (10^6^–10^1^ copies/assay) of the DNA template was used for the generation of the standard curve and served as positive control on each 96-well plate. Four smoothie samples were spiked with a known number of *Acinetobacter baylyi* cells to determine DNA recovery rates from these samples.

In total, 9 measurement points per sample (3 biological replicates with 3 technical replicates, respectively) were generated.

Cp-values were calculated using the second derivative maximum algorithm of the LightCycler 480 Software v 1.5.0. Absolute quantification results are reported as target copies per milliliter smoothie (c/mL) or per gram produce (c/g).

Relative abundances were calculated using the delta CT procedure by utilizing 16S rRNA gene crossing points as the reference target for the ARG and MGE ratios [36].

### 2.4. Microbiome and Co-Occurrence Network Analysis

The microbiome of the smoothie and produce samples was determined by paired-end sequencing the V3-V4 region of the 16S rRNA gene using the 600-cycle MiSeq reagent Kit v3 on the MiSeq platform (Illumina, San Diego, CA, USA). The library preparation was performed according to the Illumina 16S Metagenomic Sequencing Library Preparation protocol using the recommended primer sequences [37]. Smoothie samples were sequenced in biological triplicates, whereas one biological replicate was sequenced for each fresh produce sample.

The bioinformatic processing incorporated the trimming of primers via cutadapt. Filtering, denoising, merging of paired-end reads and chimera removal were performed using the DADA2 package in RStudio 2022.12.0+353 using R 4.3.3. Taxonomy was assigned to the obtained amplicon sequence variants (ASVs) using the SILVA v138.1 Ref NR 99 SSU database. Triplicates were summed and presented as a single sample for bacterial community composition analysis. Phyloseq was used to gather information based on microbial diversity using α- (Observed, Chao1, Shannon, Simpson and Inverse Simpson) and ß-diversity (Bray–Curtis dissimilarity depicted in NMDS-plots) indices. The R package ggplot2 and Microsoft Excel 2016 were used for visualization of the results.

ASVs were filtered for 0.1% read abundance meaning that the number of reads per ASV in a sample was divided by the total number of generated reads per sample. ASVs with a percentage of 0.1% and above were considered for bacterial community analysis.

The WHO bacterial priority pathogens list 2024 was used to find potential pathogens among the sequenced genera [38].

For the identification of naturally transformable bacteria (i.e., bacterial species and genera that have been shown to develop competence for the uptake of free extracellular DNA from their environment under naturally occurring conditions and not by artificial measures like heat shock, high concentrations of cations, electroporation and/or other similar unphysiological procedures), PubMed and SCOPUS were screened using the procedure as delineated in [39]. The obtained list of naturally competent bacterial genera and species was updated in February 2023. A Spearman’s rank correlation analysis was performed by combining the absolute quantification results generated via qPCR with the obtained genera filtered for 0.1% abundance in R using the “qqpubr” package. The obtained correlation matrix was filtered for strong correlations with a Spearman’s rank above 0.65 and an adjacency table was created using the “igraph” R package. A co-occurrence network analysis was created using the “igraph” and “visNetwork” R packages.

### 2.5. Statistical Analysis

All statistical analyses were performed using R Statistics version 4.3.3 (https://www.R-project.org/, accessed on 10 March 2024) unless otherwise stated.

For qPCR assay validation semi-logarithmic dilution series of target amplicons were tested (8000–0.5 copies per 2 µL). Each dilution step was carried out using 24 replicates, generating a total of 168 data points for the statistical analysis per assay. Limits of detection (LODs) with a 95% positive call rate per target were estimated using a probit generalized linear mixed model with 1000 bootstrapping replications in R Studio 2021.09.4 (R.4.1.2.). For the determination of the linear fit of the calibration curves, Pearson’s linear regression coefficients were calculated, and for estimating the repeatability and reproducibility of the assays, intra- and inter-assay variabilities were computed using arithmetic means, median, standard deviations, coefficients of variation and minima and maxima in Excel 2016. The results were also visualized with this software package. Relative abundances were calculated as ratios of ((ARG target)/(16S rRNA gene)) using the delta CT procedure and 16S rRNA gene crossing points as the reference target for the ARG and MGE ratios [36]. For more details on qPCR assay validation, see Section 2.3 and the Supplementary Materials.

The bioinformatic processing of Illumina MiSeq data for microbiome characterization was performed using the DADA2 package in RStudio 2022.12.0+353. Microbial alpha and beta diversity metrics were obtained using the phyloseq package in R. Results were visualized with ggplot2 and Excel 2016.

For co-occurrence network analysis, a Spearman’s rank correlation analysis was performed by combining absolute qPCR results with genera filtered for ≥ 0.1% abundance in R using the “qqpubr” package. The obtained correlation matrix was filtered for strong correlations (≥ 0.65). The results were visualized using the “igraph” and “visNetwork” R packages.

A one-tailed paired samples Student’s *t*-test (α = 0.05) was applied to detect differences in the mean ARG prevalences between untreated fresh vs. pasteurized/HPP-treated smoothies. The effect size “d” of the treatment on ARG prevalences was determined according to Cohen. For the interpretation of the effect size the following scheme was applied: small (d ≥ 0.2), medium (d ≥ 0.5) and strong (d ≥ 0.8). Details on the applied procedures can be found in the Supplementary Materials.

## 3. Results

### 3.1. Prevalence and Absolute Abundance of Clinically Relevant Antibiotic Resistance Genes in Smoothies and Fresh Produce

A total of 14 freshly prepared, 2 cold-pressed, 2 pasteurized and 2 high-pressure-processed smoothies as well as 2 conventionally and 2 organically cultivated carrots, lettuce and tomato samples were collected and analyzed for ARGs and MGE-associated genes using probe-based qPCR.

In the pool of twenty smoothie samples, 20 different ARGs and MGEs (from a total of 26 resistance-associated targets analyzed) could be identified per sample (Figure 1). Five to sixteen targets were observed per sample. The lowest number of different ARG and MGE targets were detected in HPP samples (5–7), followed by pasteurized smoothies (6 and 7 detected targets); the two cold-pressed samples contained 9 and 11 and the 14 freshly prepared beverages contained 6–16 different ARGs and MGEs. The 12 fresh produce samples displayed a total of 12 different ARGs and MGEs with a prevalence of 1–10 per sample, detecting 1–3 MGEs and ARGs in tomatoes, 7–9 in carrots and 8–10 in lettuce.

Targets below the limit of detection and therefore considered negative in any smoothie and fresh produce sample tested were *qnrS* (resistance against fluoroquinolones), *vanA* (vancomycin), *sat-4* (streptothricin) and the genes inactivating the β-lactamases *bla_KPC_*, *bla_NDM-1_* and *bla_OXA-10_*.

The following targets were present in all 20 beverages: the 16S rRNA gene, the insertion sequence *ISPps* and the class 1 integron-integrase gene *intI1*. Targets appearing in more than 50% of all beverages were the antimicrobial resistance genes *qacEΔ1* (inactivating biocides; present in 95% of the samples), *bla_TEM-1_* (β-lactams; 85%), *ermB* (macrolides; 75%), *sul1* (sulfonamides; 75%), *cmxA* (chloramphenicol; 70%), *tet(M)* (tetracyclines; 65%) and *strB* (aminoglycosides; 55%).

Equal to or being present in less than 50% of all smoothie samples were the targets *bla_CTX-M-1-15_* (β-lactams; 50%), *aadA* (aminoglycosides; 40%), *aph(3′)-IIa* (aminoglycosides; 30%), *ermF* (macrolides; 25%), *tet(W)* (tetracyclines; 20%), *aph(3′)-IIIa* (aminoglycosides; 15%), *dfrA-1* (trimethoprim; 15%), *tet(A)* (tetracyclines; 15%), *mcr-1* (colistin; 10%), *mecA* (β-lactams; 10%) and *tet(O)* (tetracyclines; 10%). The *t*-test result comparing the mean ARG prevalences of untreated and treated (HPP/pasteurized) smoothies confirmed with high confidence (*p* = 2.32 × 10^−4^) that the observed differences were not caused randomly and that the impact of treatment (d = 1.48) was strong and significantly reducing ARG prevalences in the HPP/pasteurized cohort (Supplementary Materials).

Concerning fresh produce, lettuce and carrot samples showed comparable ARG and MGE prevalences. The 16S rRNA gene and *ISPps* targets were present in all three types of produce (i.e., carrots, lettuce and tomatoes) (Figure 1). *IntI1* and *aadA* were detected in all carrot and lettuce samples, while only one organic tomato tested positive for both targets. In tomatoes, all remaining targets were below the LOD (Figure 1). All lettuce and carrot samples tested positive for the targets *qacEΔ1*, *ermB*, *aadA* and *strB*. Targets that were not found in all carrot and lettuce samples were *tet(M)* (detection rate in tested samples: 87.5%), *cmxA* (75%), *sul1* (50%), *aph(3′)-IIIa* (25%), *tet(A)* (25%) and *dfrA-1* (12.5%).

All β-lactam inactivating genes (*bla_CTX-M-1-15_*, *bla_KPC_, bla_NDM-1_, bla_OXA-10_, bla_TEM-1_* and *mecA*), as well as a*ph(3′)-IIa*, *ermF*, *mcr-1*, *mecA*, *qnrS*, *sat-4, tet(O)*, *tet(W)* and *vanA* were below the LOD in all carrot, lettuce and tomato samples (Figure 1).

The highest absolute concentrations in the positive tested smoothie samples were observed for *bla_CTX-M-1-15_* (1.2 × 10^5^ c/mL, in sample S9) and *strB* (6.3 × 10^4^ c/mL, S9), followed by *qacEΔ1* (5.4 × 10^3^ c/mL, S9), *ermF* (7.2 × 10^3^ c/mL, S9) and *aadA* (1.0 × 10^4^ c/mL, S15) (Figure 1). Both MGEs also showcased concentrations above 10^4^ copies per mL in the twenty smoothie samples: *ISPps* with 1.5 × 10^4^ c/mL in S9 and *intI1* with 1.6 × 10^4^ c/mL in S6. Individual samples with the highest summed ARG and MGE concentrations were the freshly produced beverages S9 (2.2 × 10^5^ c/mL), S15 (9.7 × 10^4^ c/mL), S6 (6.9 × 10^4^ c/mL) and S14 (6.5 × 10^4^ c/mL). The lowest summed ARG and MGE concentrations were observed in HPP (4.1 × 10^3^ c/mL and 5.2 × 10^3^ c/mL) and pasteurized samples (4.4 × 10^3^ c/mL and 5.9 × 10^3^ c/mL) as well as freshly prepared sample S7 (4.2 × 10^3^ c/mL).

Among the fresh produce samples, the conventionally grown lettuce samples had the highest ARG and MGE abundances (5.2 × 10^4^ c/g in sample C1 and 7.6 × 10^4^ c/g in C2), followed by the organically fertilized carrot O1 (2.4 × 10^4^ c/g), conventionally grown carrot C1 (2.1 × 10^4^ c/g) and organically cultivated lettuce samples (2.1 × 10^4^ c/g in O1 and 2.0 × 10^4^ c/g in O2). The MGE *ISPps* showed the highest overall concentrations, and the highest abundance observed in the conventionally farmed lettuce sample C1 with 3.4 × 10^4^ c/g. Similarly, the highest *intI1* concentration was found at 2.1 × 10^4^ c/g in the conventionally grown lettuce sample C2.

Individual targets with the highest concentrations were *qacEΔ1* (2.1 × 10^4^ c/g), *strB* (1.2 × 10^4^ c/g) and *aadA* (7.5 × 10^3^ c/g), all found in the conventionally grown lettuce sample C2. The carrot O1 had the highest *tet(M)* (9.1 × 10^3^ c/g) and *cmxA* (5.1 × 10^3^ c/g) concentrations, whereas *dfrA-1* only occurred in the lettuce C2 with 3.8 × 10^3^ c/g.

The 16S rRNA gene concentrations in the beverages ranged from 2.0 × 10^3^ to 6.8 × 10^8^ c/mL, with the lowest 16S rRNA gene concentrations found in HPP samples (2.0 × 10^3^ c/mL and 2.4 × 10^3^ c/mL), followed by pasteurized (8.6 × 10^3^ c/mL and 5.1 × 10^6^ c/mL), cold-pressed (2.5 × 10^6^ c/mL and 8.7 × 10^6^ c/mL) and freshly prepared samples (arithmetic average of 1.8 × 10^8^ c/mL). Fresh produce samples showcased high 16S rRNA gene copy numbers between 4.2 × 10^7^ c/g to 1.5 × 10^10^ c/g, with the lowest 16S rRNA gene abundances found in tomatoes, while the highest concentrations were observed in the lettuce samples.

The DNA yield was measured spectrophotometrically and ranged from 216.6 ng/mL–2133 ng/mL. Freshly prepared samples had on average 2–4 times higher DNA yields than HPP, pasteurized- and cold-pressed samples.

### 3.2. Bacterial Composition Analysis of Smoothies and Fresh Produce Samples

The 16S rRNA gene regions V3-V4 were amplified and sequenced for all three biological replicates of freshly prepared and cold-pressed smoothie samples as well as for the first biological replicate of all 12 fresh produce samples. The two HPP and two pasteurized samples did not pass the library preparation due to insufficient amounts of amplifiable DNA.

Between 74,210 and 177,306 high-quality filtered reads were obtained for the 3 × 16 sequenced smoothie samples. When combining the three replicates for each sample, a minimum read depth of 299,827 up to 515,241 reads was allocated to the samples. After removing mitochondrial- and chloroplast-derived sequence contaminations, between 12,552 and 59,337 microbial reads remained per replicate or a combined triplicate sum of a minimum 39,213 to a maximum of 162,848 reads was used for further analysis. To compare the samples with varying sequencing depths, relative read abundances were calculated per amplicon sequence variant (ASV) using the cleaned total read counts. Only relative read counts equal to or greater than 0.1% were included in the analysis.

**Figure 2:**
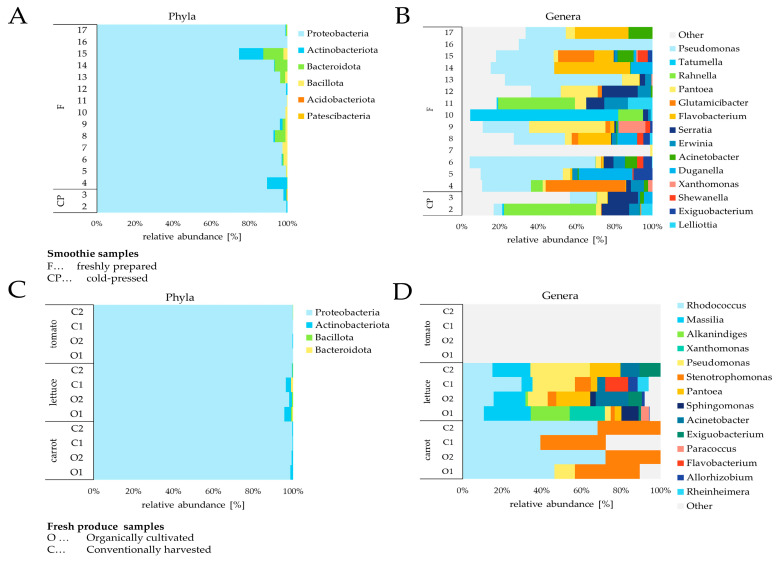
Bacterial community composition of smoothies (**A** phyla, **B** genera) and fresh produce (**C** phyla, **D** genera) represented as 100% stacked bar charts. Taxa with a relative read abundance ≥ 0.1% are displayed. The 14 most abundant genera are plotted. The remaining assigned genera are summarized under the term “Other”.

Following these criteria, a total of six phyla remained among the freshly prepared and cold-pressed smoothies (Figure 2). These were dominated by *Proteobacteria* (74.5–100%) followed by *Actinobacteriota* (0.1–12.7%), *Bacteroidota* (0.03–10.6%), *Bacillota* (former *Firmicutes*, 0.05–2.6%) as well as *Acidobacteriota* (0.1%) and *Pastescibacteria* (0.1%), which were only present in the cold-pressed sample 3. Non-assigned ASVs made up between 0.04 and 0.5% of the relative read abundance per sample.

After filtering, a total of four distinct phyla remained in fresh produce. Similar to the smoothie samples, *Proteobacteria* was the dominant phylum in all carrot, lettuce and tomato samples with relative read abundances of 95.4–100.0%. The remaining phyla *Actinobacteriota*, *Bacillota* and *Bacteroidota* had read abundances in the ranges of 0.3–3.3%, 0.1–0.6% and 0.4–1.0%, respectively, but were not present in all three produce types. *Actinobacteriota* were not detected in both conventionally and organically farmed tomato O1. *Bacteroidota* were only detected in two lettuce samples, while *Bacillota* occurred in 3 lettuce and 1 conventionally farmed tomato samples. Non-assigned ASVs on the phylum level had a relative read abundance of 0.2–0.5%.

The taxonomic rank “genus” presented diverse taxa compositions in the smoothie samples with 99 different genera ≥ 0.1% in relative read abundance.

The 14 most abundant genera were *Tatumella* (0.04–57.0%), *Pseudomonas* (0.2–28.4%), *Rahnella* (0.1–25.8%), *Pantoea* (0.04–14.5%), *Glutamicibacter* (0.0–11.0%), *Serratia* (0.1–7.7%), *Flavobacterium* (0.0–6.5%), *Xanthomonas* (0.04–5.2%), *Acinetobacter* (0.03–4.9%), *Lelliottia* (0.1–3.2%), *Shewanella* (0.04–3.0%), *Erwinia* (0.1–2.9%), *Duganella* (0.04–2.6%) and *Exiguobacterium* (0.4–1.9%). The remaining 85 genera constituted a relative read abundance between 0.5 and 10.8% per sample. ASVs that could not be assigned to a distinct genus were in the range of 26.3–98.5% per sample. The freshly prepared smoothies 5, 7, 11, 12, 16 and 17 contained over 90% non-assigned ASVs on the genus level.

Thirty distinct genera were detected in the 12 fresh produce samples with a relative read abundance ≥ 0.1% (Figure 2).

The 14 most abundant genera in produce samples were *Massilia* (0.4–6.1%), *Alkanindiges* (0.1–5.1%), *Xanthomonas* (4.6%, only present in lettuce O1), *Rhodococcus* (0.3–2.8%), *Sphingomonas* (0.2–2.2%), *Pseudomonas* (0.3–1.8%), *Pantoea* (0.3–1.5%), *Acinetobacter* (0.2–1.5%), *Paracoccus* (1.0%, only present in lettuce O1), *Flavobacterium* (1.0%, only present in lettuce C1), *Stenotrophomonas* (0.2–0.9%), *Exiguobacterium* (0.2–0.6%), *Allorhizobium* (0.1–0.4%) and *Rheinheimera* (0.5%, only present in lettuce C1). The remaining genera made up a relative abundance of 0.1–1.4% per sample. Tomato samples O1 (organically farmed) and C1 (conventionally grown) had no ASVs assigned to a specific genus considering a relative read abundance ≥ 0.1%. The conventionally farmed tomato C2 had only *Bacillus* assigned on genus level with 0.1%, while the organic tomato O2 had only *Corynebacterium* with 0.3%. Tables S2 and S3 summarize the relative read counts with abundances ≥ 0.1% detected per sample for the taxonomic ranks genus as well as species. On the species level, *Tatumella punctata*, *Pseudomonas azotoformans*, *Glutamicibacter arilaitensis*, *Xanthomonas citri* and *Pantoea vagans* presented the highest relative read abundances of assigned ASVs. However, most ASVs (22.6–83.4% for smoothies, 69.8–98.6% for fresh produce) could not be assigned on the species level despite relative read abundances ≥ 0.1%.

In terms of bacterial diversity, the richness indices of Observed ASVs and Chao1 showed nearly identical results with the highest average ASV count in cold-pressed (179 ± 150 ASVs) and freshly- prepared smoothies (150 ± 150 ASVs), followed by organically (77 ± 18 ASVs) and conventionally cultivated lettuce (67 ± 21 ASVs), organic (42 ± 13 ASVs) and conventionally grown carrots (32 ± 6 ASVs) and organically (30 ± 9 ASVs) and conventionally grown tomatoes (28 ± 4 ASVs). The diversity metrics Shannon showed the lowest diversity in lettuce samples irrespective of their harvesting status, while the freshly prepared and cold-pressed smoothies were the most diverse. The evenness indices Simpson and inverse Simpson supported these results (see Supplementary Materials, Figure S1).

To showcase the differences between all samples, a Bray–Curtis dissimilarity was calculated and plotted using non-metric multidimensional scaling (NMDS) ordination (Figure S2). All smoothies, irrespective of being cold-pressed or freshly prepared, were clustering closely together with higher dissimilarities in samples S17 and S16. Fresh produce samples were clearly distinguished as distinct clusters separated by a higher dissimilarity from the smoothie samples. The cultivation status did not impact the clustering of each vegetable. Tomato samples had the highest dissimilarity score and clustered around NMDS1 −6.5 and NMDS2 −0.5.

For the identification of potential pathogens in the tested food samples, the WHO bacterial priority pathogens list was consulted [38]. Considering taxa with relative abundances ≥ 0.1%, the order *Enterobacterales* was found with 20 different genera in smoothies and 5 in the carrot and lettuce samples. The following *Enterobacterales* genera (also comprising potential pathogens) were identified in smoothies: *Aeromonas*, *Alishewanella*, *Arsenophonus*, *Buttiauxella*, *Citrobacter*, *Enterobacter*, *Erwinia*, *Escherichia*, *Klebsiella*, *Lelliottia*, *Pantoea*, *Pectobacterium*, *Rahnella*, *Raoultella*, *Rheinheimera*, *Rosenbergiella*, *Serratia*, *Shewanella*, *Tatumella* and *Yersinia*. In the fresh produce samples, the genera *Kosakonia*, *Pantoea*, *Rahnella*, *Rheinheimera* and *Siccibacter* were found. Additionally, *Streptococcus salivarius* was also detected in fresh produce sample 8 with an average relative abundance of 0.5%. Figure S3 presents the average relative read abundances per sample categorized in order, genus and species (if available). None of the other priority bacterial pathogens were identified in the tested samples. 

### 3.3. Detection of Naturally Transformable Bacteria

Additionally, our sequencing data were screened for bacteria naturally capable of taking up extracellular DNA (potentially encoding ARGs). The sequence data package was filtered for read counts above 10 rather than for relative read abundances ≥ 0.1%. A total of 10 different genera and 19 distinct species reported in the scientific literature to become competent for DNA uptake under naturally occurring environmental conditions were detected in 13 smoothies and the two conventionally grown lettuce samples with overall very low relative read abundances (0.0001–0.006%) (Table S4). Freshly prepared smoothie sample S8 had the highest count of bacteria categorized as naturally transformable (*Acidovorax* spp., *Acinetobacter calcoaceticus* and *Aeromonas* spp.). *A. calcoaceticus* had the overall highest relative read abundance (0.006%) and was found in the lettuce sample C2. The bacterium was also detected with the highest relative read abundance in the cold-pressed smoothie S3 (0.0025%), while the genus *Acidovorax* and *Aeromonas veronii* had the highest relative read abundances in freshly prepared smoothies (0.0035% and 0.003%, respectively).

### 3.4. Co-Occurrence Network Analysis of Bacterial Genera and ARGs

To identify potential ARG carriers in the characterized bacterial communities, a network analysis was performed trying to establish correlations between ARGs and the assigned genera (relative read abundance ≥ 0.1%). A Spearman’s correlation coefficient of ≥0.65 was selected as the cutoff limit to filter for strongly correlating ARGs and MGEs with each other as well as with assigned genera.

**Figure 3:**
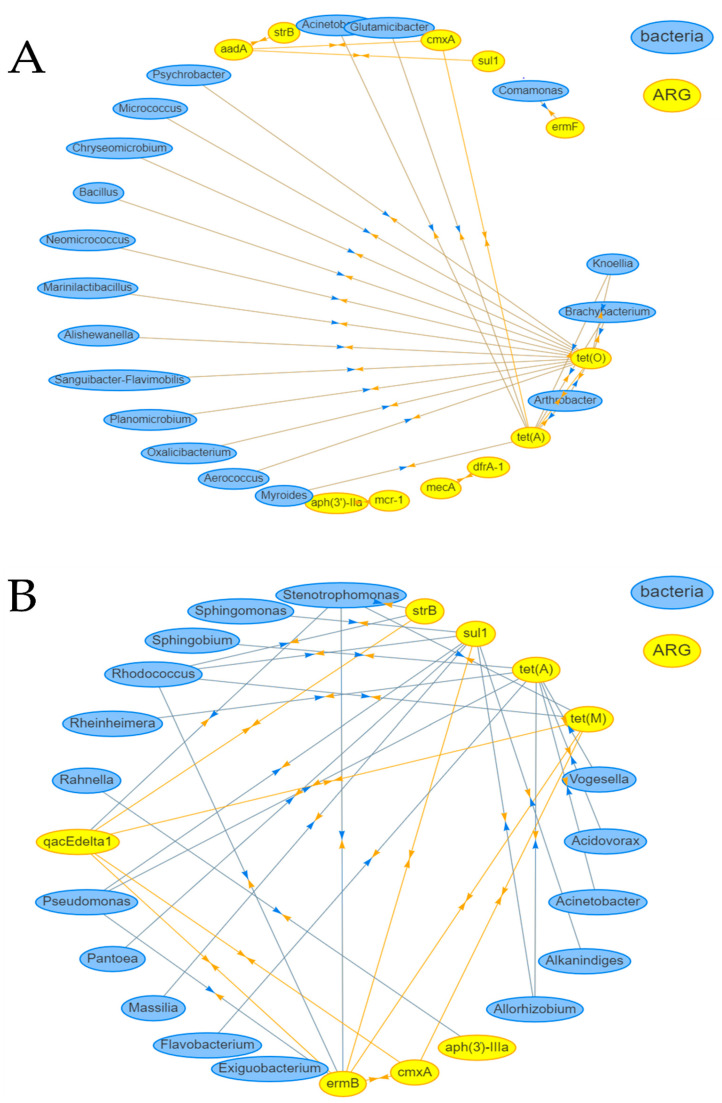
Co-occurrence network analysis of genera and absolute ARG and MGE concentrations (Spearman’s correlation coefficients ≥ 0.65) for (**A**) smoothies and (**B**) fresh produce.

Using these filtering steps, smoothie samples had 30, while fresh produce samples had 23 strongly correlating items. The strongest and most abundantly correlated target was the tetracycline resistance gene *tet(O)* in smoothies (Figure 3). This ARG was positively correlated with *Brachybacterium* and *Knoellia* with a Spearman’s correlation coefficient (ρ) of 1. Other positively correlating items were the tetracycline resistance gene *tet(A)* and *Arthrobacter* (ρ = 0.84, respectively) and the genera *Planomicrobium*, *Neomicrococcus*, *Alishewanella*, *Chryseomicrobium*, *Oxalicibacterium*, *Bacillus* and *Psychrobacter* (ρ = 0.73, respectively). The ARG *tet(A)* was positively correlated with the genera *Knoellia* and *Brachybacterium* (ρ = 0.84, respectively), *Myroides* (ρ = 0.79), *Arthrobacter*, *Acinetobacter*, *Glutamicibacter* and the phenicol resistance gene *cmxA* (ρ = 0.67, respectively). Several ARGs also positively correlated with each other like the aminoglycoside resistance gene *aadA* with another aminoglycoside resistance gene *strB* (ρ = 0.72), *cmxA* (ρ = 0.68) and the sulfonamide resistance gene *sul1* (ρ = 0.67). The trimethoprim resistance gene *dfrA-1* positively correlated with the β-lactam resistance gene *mecA* (ρ = 0.84) as well as the colistin resistance gene *mcr-1* with the aminoglycoside resistance gene *aph(3′)-IIIa* (ρ = 0.84). The macrolide resistance gene *ermF* positively correlated with *Comamonas* (ρ = 0.65). The only strongly negatively correlating items were the biocide resistance gene *qacEΔ1* with *Allorhizobium/Neorhizobium/Pararhizobium/Rhizobium* (ρ = 0.65). We found 17 distinct genera that correlated with ARGs with variable read abundances in the tested smoothie samples, while only sample 15 contained all of these genera.

*Tet(A)* showed the highest correlation scores in vegetable samples. It positively correlated with the genera *Allorhizobium/Neorhizobium/Pararhizobium/Rhizobium* and *Acinetobacter* (ρ = 0.85, respectively), *Sphingobium*, *Flavobacterium*, *Acidovorax*, *Rheinheimera*, *Vogesella* (ρ = 0.74, respectively) and *Pseudomonas* (ρ = 0.73). *CmxA* correlated positively with *qacEΔ1* (ρ = 0.81), *ermB* (ρ = 0.78) and *tet(M)* (ρ = 0.68). Besides *cmxA*, the macrolide resistance gene *ermB* also positively correlated with *Rhodococcus* (ρ = 0.89), *tet(M)* (ρ = 0.81), *Stenotrophomonas* (ρ = 0.79), *Pseudomonas* (ρ = 0.76), *qacEΔ1* (ρ = 0.68) and *sul1* (ρ = 0.66). Besides the ARGs *cmxA* and *ermB*, the biocide resistance gene *qacEΔ1* also correlated with *tet(M)* and *Stenotrophomonas* (ρ = 0.81, respectively), as well as the ARG *strB* (ρ = 0.68). *StrB* also correlated with *Rhodococcus* (ρ = 0.88) and *Stenotrophomonas* (ρ = 0.85). *Sul1* positively correlated with *Allorhizobium/Neorhizobium/Pararhizobium/Rhizobium* (ρ = 0.86), *Massilia* (ρ = 0.80), *Alkanindiges* and *Sphingomonas* (ρ = 0.79, respectively), *Rhodococcus* (ρ = 0.77), *Pantoea* (ρ = 0.74) and *Pseudomonas* (ρ = 0.69). *Tet(M)* positively correlated with the genera *Stenotrophomonas* (ρ = 0.86) and *Rhodococcus* (ρ = 0.68).

When comparing the results of the network analysis and the bacteria overlapping with the bacterial priority pathogen list of the WHO, only the bacterium *Alishewanella* of the order *Enterobacterales* was found to correlate with the tetracycline resistance gene *tet(O)*. However, the order *Enterobacterales* is categorized as “critical” only in combination with third-generation cephalosporin- or carbapenem resistance [38].

## 4. Discussion

In tune with the One Health initiative where the threat of AMR along the human, animal and environment sectors is highlighted, this study aimed to evaluate differently processed smoothies and fresh produce as a potential environmental source of ARGs and MGEs. The qPCR approach revealed the highest prevalence of ARGs in freshly prepared smoothies with the MGEs *ISPps* and *intI1* as well as the ARGs *qacEΔ1* (inactivating biocides), *bla_TEM-_*_1_ (β-lactams like penams, penems, cephalosporins and monobactams), *ermB* (macrolides), *sul1* (sulfonamides), *cmxA* (chloramphenicol), *tet(M)* (tetracyclines) and *strB* (aminoglycosides) being present in more than 50% of the smoothies. With the exception of *bla_TEM-1_*, these targets were also the most abundant in carrots and lettuce samples, highlighting the potential of foodstuff for being a reservoir of ARGs and MGEs. These findings are in accordance with studies conducted with fresh produce in Switzerland, where 95% of the tested samples (including tomatoes, lettuce, strawberries, coriander and carrots) were positive for ARGs such as *bla_TEM-1_*, *ermB* and *sul1* but also the integron-integrase class 1 gene *intI1* [16]. These resistance determinants can be transferred intracellularly via ARB or encoded on free extracellular DNA, especially if the drinks are consumed without prior treatment (e.g., by pasteurization or HPP).

The presence of *intI1* in all our smoothie, carrot and lettuce samples is also in agreement with Mohamed et al., who reported class 1 integrons in 100% of the *E. coli* isolates from ready-to-eat vegetables (lettuce, beans, cabbage, etc.) [40]. In addition, the *intI1* gene was detected in the total DNA from all produce samples (mixed salad, arugula, and cilantro) collected in a study from Germany but was only present in 27% and 36.5% of the *sul1* and *qacEΔ1* positive *E. coli* isolates [41]. The highest *intI1* concentrations in our analysis were observed in a smoothie (1.6 × 10^4^ c/mL) and in lettuce (2.1 × 10^4^ c/g). Zhao et al. reported the presence of *intI1* in endophytes of carrots, tomatoes, lettuce, cucumber and cabbage at concentrations between 3.1 × 10^5^ (cabbage)–1.2 × 10^8^ c/g (cucumber) [42]. These data indicate a broad variety of *intI1* prevalences and concentrations in plant-based food according to the matrix analyzed and to the anthropogenic activities (i.e., selection pressure) these specimens have been exposed to over their lifecycle along the food chain [43].

Variabilities were also encountered in tomatoes, where from 24 ARGs, only the aminoglycoside resistance gene *aadA* was detected in 25% of tomatoes, while the MGE *ISPps* was found in all tested samples, suggesting the potential for HGT in these tomato-borne bacteria. This result stands in contrast to Kläui et al., who reported the presence *of vanA*, *tet(W)*, *qnrS*, *ermB*, *sul1* and *bla* genes in at least 83% of the tested tomatoes [16]. Similarly, a study by Cerqueira et al. revealed ARG prevalences in Spanish tomatoes of 100% for *bla_TEM_*, 88% for *sul1*, 38% for *qnrS*, 75% for *tet(M)* and 38% for *mecA*.

The following specific conditions might provide an explanation for the observed differences: (i) location where the samples were collected (high- vs. low-income countries), (ii) applied fertilization (e.g., manuring) and irrigation (e.g., reclaimed wastewater) procedures performed during cultivation of the crops, (iii) proximity to soil during cultivation, (iv) crop type and (v) sampling procedure: analysis of total DNA vs. selective enrichment and/or pre-selection of certain bacterial carrier species followed by analysis of cultivated clones but also the limited sample size in our pilot study.

While tomatoes represented an outlier in our pilot study with very low ARG abundances and microbial burden, carrots and lettuce were comparable to the Swiss study, where *intI1*, *sul1*, *aadA*, *ermB*, *tet(W*) and *bla* genes were detected [16].

When looking at ARGs in terms of public health, resistances obtained by *E. coli* and bacterial species classified as ESKAPE pathogens (*E. faecium, S. areus*, *K. pneumoniae, A. baumannii*, *P. aeruginosa* and *Enterobacter* spp.) pose the highest risk for therapeutic failure. Depending on the organism, (amino-)penicillins, third-generation cephalosporins, carbapenems, fluoroquinolones, aminoglycosides, macrolides, vancomycin and rifampicin were the most prescribed anti-infective drug classes between 2020-2022 according to EARS-net [44]. In our study, we detected several ARGs capable of inactivating some of these antibiotics, such as the extended-spectrum β-lactamase (ESBL) gene *bla_CTX-M-1-15_* (effective against, e.g., third-generation cephalosporins used against *E. coli*, *K. pneumoniae*, *S. pneumoniae* and *P. aeruginosa*), *ermB* (resistance against macrolide antibiotics that are used for *S. pneumoniae* infections), *bla_TEM-1_* (resistance against aminopenicillins used for *E. coli* infections) and *vanA* (resistance against vancomycin used for *E. faecium* infections) [44].

The high prevalence of the sul1 and *intl1* genes also indicates extensive anthropogenic contamination, as they were present in lettuce and freshly prepared, cold-pressed and pasteurized smoothies [45]. 

A critical result of our pilot study was the high abundance of the β-lactamase gene *bla_CTX-M-1-15_* in freshly prepared and cold-pressed smoothies. ESBL-producing bacteria are of special concern in the clinical setting, as β-lactam agents are the most prescribed antibiotics globally [44]. Clinically critical bacterial infections caused by *E. coli*, *K. pneumoniae* and *S. pneumoniae* are mainly treated by antimicrobial agents such as ceftriaxone or ceftazidime, which can be inactivated by CTX-M beta-lactamases [44]. These CTX-M ESBLs have rapidly evolved via HGT, underlining the importance of reducing the spread of these genes [46].

CTX-M ESBLs were also detected in Swiss ready-to-eat diced tomatoes, chopped chives and recycled wash water for irrigation in isolates of *Kluyvera ascorbata* and *E. coli*, highlighting a contamination risk in freshly prepared smoothies [47].

The aminoglycoside resistance gene *strB* was also detected in freshly produced and cold-pressed smoothies. This ARG is known to inactivate streptomycin, a formerly essential first- and later on, a second-line antibiotic used against, e.g., tuberculosis caused by *Mycobacterium tuberculosis* and is found completely or partially on gene cassettes [48]. Besides its clinical usage, streptomycin was also used for a long time for the treatment of fire blight found in pome fruit. Due to the antibiotic’s decade-long widespread usage in agriculture, resistances were acquired by plant-pathogenic bacteria [49]. *StrB* was also found at elevated concentrations in one of the conventional salad samples together with *intI1*, *ISPps* and *qacEΔ1*, further emphasizing the mobility of ARGs using MGE-associated means.

Tomatoes showed comparable 16S rRNA gene concentrations with freshly prepared smoothies, while carrots and salads exceeded those by 1.3 and 1.8 log_10_. Data regarding absolute 16S rRNA gene concentrations in these matrices are, however, still scarce in the scientific literature. Absolute concentrations from 10^5^ c/g up to 10^8^ c/g in lettuce and 10^7^ c/g in carrots have been reported; however data, for tomatoes and fruit juices remain limited and if available, only account for culturable bacteria using colony-forming units for quantification [16,50].

Our results also showed the dominance of the phylum *Proteobacteria* in both smoothies and fresh produce in accordance with other studies analyzing vegetable and fruit samples [51,52]. This phylum is also associated with high HGT rates and mobile ARG prevalences in agricultural soil [53,54]. Among this phylum, one of the genera was *Pseudomonas*, which is found ubiquitously in the environment and comprises well-known opportunistic pathogens like *P. aeruginosa* that have the potential to cause life-threatening, predominantly nosocomial infections in humans [55]. This pathogen has also been cultivated in a previous study from 20% of the freshly prepared smoothie samples among other genera such as *Klebsiella*, *Citrobacter*, *Enterobacter*, *Bacillus* and *Pantoea* [56]. Pseudomonads are often part of the phyllo- and rhizosphere of plants. They can be beneficial for plant growth and biocontrol abilities due to the secretion of secondary metabolites ranging from antibiotics to plant hormones [57,58]. *Pantoea* is a soil- and plant-associated bacterium that was highly abundant in our samples. Some species (e.g., *P. dispersa* and *P. agglomerans*) are known pathogens causing bloodstream infections, cholangitis or pneumonia in humans [59]. *Acinetobacter baumannii* is a perilous ESKAPE pathogen that causes severe nosocomial infections [60]. *Acinetobacter* spp. easily takes up extracellular DNA encoding resistance genes against most clinically relevant antibiotics from non-related species [61].

Using DNA-based methods, none of the bacteria listed in the WHO bacterial pathogen priority list of 2024 were observed to carry ARGs such as *bla_CTX-M-1-15_*, *bla_KPC_*, *bla_NDM-1_*, *bla_OXA-10_*, *bla_TEM-1_* and *mecA* when comparing the results of the co-occurrence network analysis.

The phyla Actinobacteriota, Bacillota and Proteobacteria showed strong correlations in our network correlation analysis for manure-associated genes such as *tet(A)* and *tet(O)* in the freshly prepared and cold-pressed smoothies, suggesting potential contamination sources for produce in the near vicinity of (manured) soil [62]. This assumption can be further expanded as our tested produce samples remained our most frequently correlated targets (*tet(A)* and *tet(M)*).

Tetracycline resistance phenotypes of the correlated genera *Acinetobacter*, *Arthrobacter*, *Brachybacterium*, *Knoellia*, *Micrococcus*, *Aerococcus*, *Bacillus*, *Planomicrobium*, *Alishewanella*, *Myroides* and *Psychrobacter* were confirmed by previous studies and found in isolates collected from, e.g., patients in intensive care units, blood culture, urinary tracts but also environmental samples like air conditioners and activated sludge around pharmacies [63,64,65,66,67,68,69,70,71,72].

The strong correlations of these tetracycline resistance genes with multiple different bacteria could be attributed to the widespread use of tetracyclines in agriculture and food animals in Europe and Austria specifically [73]. Similarly, correlations with resistance genes inactivating antibiotic classes such as macrolides (*ermB*), aminoglycosides (*strB, aph(3′)-IIIa*) and sulfonamides (*sul1*) could also be promoted by the extensive use of these antimicrobial agents in food-producing animals despite the continuous reduction in their usage since 2011 [74]. Reasons for high and diverse microbial, ARG and MGE loads as well as genus distributions in freshly prepared smoothies can encompass differences in ingredients and their respective soil/manure proximity, hygiene regulations during preparation (washing of ingredients, hygiene and health of handler, spoilage of ingredients, contaminated equipment, etc.) and storage after preparation (cooled or at room temperature) [22]. From our results regarding fresh produce, we cannot determine whether conventional or organic cultivation has a direct effect on taxon distribution, microbial diversity, ARG prevalence or ARG abundance. Nonetheless, the prevalence of resistance genes inactivating clinically important antibiotic classes in lettuce and carrots is of concern.

In lettuce and freshly prepared and cold-pressed smoothies, ten different genera were identified as competent bacteria indicating the potential for extracellular ARG transfers directly from and within these plant matrices. Out of these, *Acinetobacter calcoaceticus*, *Escherichia coli*, *Achromobacter xylosoxidans*, *Aeromonas* spp. and *Streptococcus* spp. are known to encompass clinically relevant pathogens whose ability to acquire new resistances is especially of concern [75,76,77].

Antibiotic resistance genes can also be disseminated on free extracellular DNA in the environment by competent bacteria that have the capacity to incorporate exDNA from their surroundings [78]. In line with this observation, various food matrices have been described to be conducive to natural bacterial transformation: *Bacillus subtilis*—a common food contaminant—can develop competence for exDNA uptake in milk [79]. *E. coli* was shown to take up free exDNA in various food-associated environments like tomato, carrot and other vegetable juice, soy drink, milk, supernatants of canned soybeans, cabbage, shrimps and mixes of canned vegetables [9]. Consequently, it appears that such common foodstuffs do not provide conditions that will lead to rapid degradation of exDNA [80]. Some food matrices like soymilk, tofu and fermented sausage even seem to provide protection against DNase activity and stability to free exDNA [81,82]. Fresh plant material from leaves and grains usually contains, even after mechanical treatment, DNA fragments large enough to code for functional genes. Various canola substrates like whole seeds, cracked seeds, meals and diets could be shown to contain intact plant genes [83]. On the other hand, many fruits and vegetables are characterized by highly acidic pH conditions, which induce DNA degradation by acid hydrolysis, the disintegration of cell walls and the release of DNA-destroying endonucleases [84].

Concerning the impact of food processing technologies on their potential to reduce AMR dissemination from plant-borne microbiomes, HPP treatment achieved an average 4.6 log_10_ reduction when compared to freshly prepared samples (as assessed by 16S rRNA gene concentrations). While the FDA’s guidance documents require a 5 log10 reduction in a bacterial pathogen, our result is in line with the overall microbial load determined by the surrogate marker target 16S rRNA [85]. Pasteurization, on the other hand, appeared to be not as efficient as HPP in reducing 16S rRNA gene concentrations in the tested smoothie samples. The nucleic acid disintegrating nature of HPP was observed for ARGs such as *sul1, cmxA*, *bla_CTX-M-1-15_* and *aadA* in our samples, reducing the chances of spreading resistances determinants against cephalosporins, aminoglycosides, chloramphenicol and sulfonamides. The heat-based method of pasteurization reduced the prevalence of *bla_CTX-M-1-15_*, *cmxA*, *aadA*, *aph(3′)-IIIa* and *ermF*.

Our results indicate agreement with the scientific literature that high-pressure processing—besides destroying living bacterial cells—substantially degrades DNA [24]. This treatment reduces the probability of the presence of intact full-length genes in smoothies by several orders of magnitude and has been shown to be a promising technology in comparison to thermal processing for ensuring microbial safety and maximum retention of nutrients [86]. HPP thus appears to be the method of choice for diminishing the AMR dissemination potential from plant-based food drinks like smoothies.

## 5. Conclusions

This research is a pilot study that provides a snapshot view of the ARG, MGE as well as microbial burden in smoothies and fresh produce purchased in Austria. In minimally processed beverages such as smoothies as well as fresh produce (e.g., lettuce and carrots), high ARG, MGE and microbial diversities were observed. Class 1 integron-integrase and ISPps genes were detected in all smoothies: The presence of these MGEs indicates an increased potential for HGT (including ARGs) if smoothie-borne bacterial carriers of these genes encounter the human microbiome. The high prevalence of the class 1 integron-associated ARGs *sul1* and *qacEdelta1* in the tested samples supports this conclusion. Our data confirm the presence of various ARGs inactivating nine different clinically important antibiotic classes in smoothies with high abundances of genes inactivating cephalosporins (*bla_CTX-M-1-15_*), streptomycin (*strB*), biocides (*qacEdelta1*) and tetracyclines (*tet(M)*) highlighting the relevance of this study for public health aspects. 16S rRNA gene quantifications indicate a deleterious effect on bacterial DNA concentrations proportional to the DNA-degrading intensity potential of the applied processing procedure culminating in a DNA loss of five orders of magnitude in high-pressure processed smoothies. These observations are in agreement with the mechanisms and impacts as described in the scientific literature for these food preservation techniques and support our initial hypothesis that DNA-targeting processing will reduce smoothie-borne ARG exposure rates of the microbiome of the consumer. Our results indicate a reduction in ARG prevalences by pasteurization and HPP. Especially HPP treatment of smoothies appears to be a promising tool for reducing ARB and ARG loads and, consequently, smoothie-borne ARG exposure rates of the consumers’ microbiome. A limitation of the presented study is its small number of tested samples. Lacking qualitative and quantitative AMR data in minimally processed produce and related products requires suitable monitoring methods that capture ARG prevalence, abundance and dissemination in directly consumed products. Our data can be used as reference points for risk management to support product safety and the high-quality standards as laid down in the European food legislative framework, both for protecting the individual consumer as well as for public health.

## Figures and Tables

**Table 1:** Plant-based ingredients, providers and preservation methods of 20 smoothie samples.

Sample Number	Preservation	Main Ingredients (Decreasing Concentrations)	Provider
S1	HPP ^1^	apple, banana, spinach, pear, kale, ginger, matcha	supermarket 1
S2	cold pressed	beet, carrot, apple, ginger	juice bar 1
S3	cold pressed	fennel, green apple, cucumber, ginger, lemon, parsley, mint	juice bar 2
S4	fresh	beet, apple, orange	juice bar 2
S5	fresh	orange, apple, ananas, pear, spinach, mint, lime	supermarket 1
S6	fresh	apple, avocado, spinach, lemon	juice bar 3
S7	fresh	beet	juice bar 2
S8	fresh	orange, cabbage, cucumber, cabbage, lamb’s lettuce, banana	supermarket 1
S9	fresh	orange, apple, ananas, avocado, rocket, pepper	supermarket 1
S10	fresh	beet, apple, carrot, ginger	juice bar 3
S11	fresh	orange, carrot, apple, banana, ananas	supermarket 1
S12	fresh	beet, orange	juice bar 1
S13	fresh	spinach, apple	juice bar 3
S14	fresh	cucumber	juice bar 2
S15	fresh	spinach, orange	juice bar 2
S16	fresh	apple	supermarket 2
S17	fresh	apple	supermarket 1
S18	pasteurized	apple, kiwi, peach, zucchini, orange, kale, spinach	supermarket 2
S19	pasteurized	apple, peach, orange, spinach, zucchini, spirulina	supermarket 1
S20	HPP	apple, spinach, ginger	supermarket 2

^1^ High-pressure processing.

## Data Availability

The original contributions presented in this study are included in this article and Supplementary Materials; further inquiries can be directed to the corresponding author. The original sequencing data presented in this study are openly available in the NCBI sequencing read archive (SRA) under the BioProject ID number PRJNA1161591.

## Supplementary Materials

The following supporting information can be downloaded at: https://www.mdpi.com/article/10.3390/foods14010011/s1, Figure S1: Alpha diversity measures of bacterial communities in smoothies and fresh produce; Figure S2: Differences between smoothie and vegetable bacterial communities based on processing status; Figure S3: Potential pathogenic bacteria regarding their taxonomic rank based on the WHO bacterial priority pathogens list of 2024; Figure S4: Relative quantification of ARGs and MGEs in smoothie and fresh produce samples; Table S1: Probe-based qPCR quality parameters; Table S2: Naturally transformable bacteria in smoothies and fresh produce. Table S3: Relative read abundances of the most abundant genera and species (top 15; [%]) in the tested fresh produce. Table S4: Naturally transformable bacteria in smoothies and fresh produce. (Reference [87] is cited in Supplementary Materials).
