# Supplementary material for: Prevalence of Antibiotic Resistance Genes in Differently Processed Smoothies and Fresh Produce from Austria
Source: Foods. 2024 Dec 25;14(1):11. doi: 10.3390/foods14010011 (PMC11720611; doi:10.3390/foods14010011)
Supplement: Supplementary file 1 [file foods-14-00011-s001.zip › foods-3277098-supplementary.pdf]

Supplementary Materials

Prevalence of Antibiotic Resistance Genes in Differently Processed Smoothies and Fresh Produce from Austria

Sonia Galazka [1,2], Valerie Vigl [1], Melanie Kuffner [1], Irina Dielacher [2], Kathrin Spettel [3,4], Richard Kriz [4,5], Norbert Kreuzinger [2], Julia Vierheilig [2,6] and Markus Woegerbauer [1,*]

1. Statistical evaluation of ARG prevalences in processed and non-processed smoothie samples:

A one-tailed paired samples t-test was performed to assess whether the prevalence of ARGs detected in freshly prepared smoothies (here: non-treated samples) was significantly higher than of that observed in processed smoothies (pasteurized and high-pressure-processed). Using the paired samples t-test (different variances) module of Microsoft Excel 365 (alpha = 0.05), the difference between the non-processed samples (arithmetic mean x=10.5, standard deviation SD= 3.16) and the processed samples (x=6.25, SD=0.96) was calculated to be significant (t(16)=4.38; p = 2.32E-04). By that high degree of significance (p= 2.32E-04), the statistical evaluation showed that the observed difference between untreated and treated cohorts was not created randomly. The effect size as calculated by Cohen's d (d= 1.48) was high. The difference between the non-processed and processed smoothie samples is large, and thus the impact of processing on ARG prevalences is statistically supported to be substantial.

2. Tables:

Table S1. Probe-based qPCR quality parameters: assay performance and validation.

n = number of technical replicates per semi-logarithmic dilution step (8 dilution steps in total). R^2 = Pearson's linear regression coefficient of determination. Slope = amplification efficiency. Intra-assay and inter-assay variability: repeatability and reproducibility of the assay (coefficient of variation (%); minimum and maximum indicated). For the determination of the analytical

assay sensitivity a 95% limit of detection (LOD) was calculated using a probit generalized linear mixed model with 1000 bootstrapping replications in R Studio 2021.09.4 (R.4.1.2.). LOD units represent copies per gram or millilitre matrix. Primer specificity of each assay was confirmed by in silico alignments of the sequences with the NCBI nucleotide BLAST suite (blastn and microbial nucleotide BLAST databases; optimized for short query sequences: megablast; https://blast.ncbi.nlm.nih.gov/Blast.cgi?PAGE_TYPE=BlastSearch&BLAST_SPEC=MicrobialGenomes). Amplicon = size of the amplicon (base pairs).

qPCR	Dye	Target	n	R^2	PCR-Efficiency (slope)	Repeatability (intra-assay variability)	Reproducibility (inter-assay variability)	Sensitivity (95% LOD c/g or mL)	Forward Primer Sequences (5′->3′)	Reverse Primer Sequences (5′->3′)	Amplicon (bp)
simplex	FAM	*sul1*	24	1.000 - 1.000	-3.506 - -3.437	0.16 - 1.31	0.36 - 1.79	265	ATTGCCGATCGCGTGAA-	CCGTTGGCCTTCCTGTAAAG	68
simplex	FAM	*tet(W)*	24	0.988 - 0.996	-3.663 - -3.302	0.40 - 3.40	1.00 - 2.84	180	GGCCCAGACCCGTATTCTGT	ACGCCAGCCTGGTCGAT	87
simplex	FAM	*vanA*	24	0.998 - 1.000	-3.415 - -3.348	0.12 - 1.51	0.72 - 2.58	760	GAAATCAACCATGTTGATGTAGCATT	CCGGACAATTCAAACAGACCTT	89
simplex	FAM	bla_{TEM-1}	24	0.999 - 1.000	-3.452 - -3.359	0.21 - 1.13	0.33 - 1.16	140	GCTTCCCGGCAACAATTAATAG	CGGCTCCAGATTTATCAGCAAT	70
simplex	VIC	*ermB*	24	0.999 - 1.000	-3.522 - -3.378	0.20 - 1.78	0.38 - 1.37	180	TCACCGAACACTAGGGTTGCT	CATTCCGCTGGCAGCTTAA	72
simplex	VIC	*intI1*	24	1.000 - 1.000	-3.449 - -3.423	0.27 - 0.97	0.30 - 1.35	190	TTCAGCACATGCGTGTAAATCAT	ATTCGAACCGTGCAGGATCT	137
simplex	FAM	*aadA*	24	0.999 - 1.000	-3.437 - -3.411	0.17 - 1.68	0.54 - 2.07	170	CGCGATTTTGCCGGTTA	CTGGCGATGAGCGAAATGTA	71
simplex	FAM	*strB*	24	1.000 - 1.000	-3.533 - -3.479	0.20 - 1.57	0.24 - 1.40	580	AACGCAGGTTGTCAAACTGACTAC	CCGAGGCATTGCTCATCATT	70
duplex	FAM	bla_{KPC}	24	0.999 - 1.000	-3.537 - -3.408	0.35 - 2.60	0.36 - 2.08	135	GAACGTGGTATCGCCGATAGA	GCCGTGCAATACAGTGATAACG	105
	VIC	$bla_{CTX-M-1-15}$	24	0.994 - 0.999	-3.554 - -3.423	0.16 - 1.40	0.21 - 1.93	430	CACTTACTTCACCCAGCCTCAAC	TTTAGCCGCCGACGCTAA	67
duplex	FAM	*tet(A)*	24	1.000 - 1.000	-3.559 - -3.463	0.16 - 0.83	0.29 - 1.76	175	CCAGCCTGACCTCGATCGT	CACCCGTTCCACGTTGTTATAG	76
	VIC	*cmxA*	24	0.997 - 1.000	-3.480 - -3.361	0.28 - 3.01	0.31 - 2.21	280	GGCGGACCGCTATTGCT	AAGCGCAACGGGATGAGAT	66
duplex	FAM	bla_{NDM-1}	24	0.998 - 1.000	-3.399 - -3.332	0.22 - 1.74	0.29 - 1.57	220	TGTCGGCATCACCGAGATT	TGGTGGCTGCCTGATCAA	62
	VIC	*dfrA-1*	24	0.996 - 1.000	-3.613 - -3.360	0.17 - 2.02	0.27 - 1.61	210	CGCAAGACTTTTGAATCAATGG	TCACTACGTTCTCATTGTCAGATGTAA	94
duplex	FAM	*ermF*	24	0.999 - 1.000	-3.732 - -3.607	0.15 - 1.29	0.33 - 2.12	640	TGATGCCCGAAATGTTCAAG	AAGGAATATTTGACACCACTTTGAAA	89
	VIC	*qacEΔ1*	24	1.000 - 1.000	-3.437 - -3.414	0.21 - 2.14	0.25 - 2.97	140	CGCATTTTATTTTCTTTCTCTGGTT	GAGTCCCGACCAGACTGCAT	73
duplex	VIC	bla_{OXA-10}	24	0.999 - 1.000	-3.521 - -3.360	0.30 - 2.22	0.27 - 2.10	140	TGAGCATCAGGTTTTCAAATGG	TGCCCCTCTTAAGGTCAAGTCT	79
	FAM	*ISPps*	24	0.997 - 0.999	-3.231 - -3.171	0.35 - 1.12	0.31 - 1.09	170	CCCCAGTGCAGACCACACT	TTGGCGTCACAGTTCTCCAG	79
duplex	FAM	*mcr-1*	24	0.999 - 0.981	-3.470 - -3.157	0.20 - 2.77	0.28 - 2.11	195	GCGCAATTTGCCGATTATAAAT	CGCGGCATTCGTTATAAGGA	73
	VIC	*qnrS*	24	0.997 - 1.000	-3.558 - -3.427	0.21 - 1.97	0.27 - 2.32	585	TGGAAACTTGCATCACGAAGAT	TTCATTGAACAGGGTGATATCGA	68
duplex	FAM	*aph(3′)-IIa (npt*II)	24	0.996 - 0.999	-3.120 - -3.033	0.23 - 2.50	0.20 - 1.88	215	GATCTCCTGTCATCTCACCTTGCT	TCGCTCGATGCGATGTTTC	129
	VIC	*aph(3′)-IIIa (npt*III)	24	0.994 - 0.999	-3.225 - -3.108	0.20 - 1.70	0.16 - 1.66	230	ACATATCGGATTGTCCCTATACGAA	TCGGCCAGATCGTTATTCAGTA	82
duplex	FAM	*mecA*	24	0.998 - 1.000	-3.596 - -3.339	0.21 - 1.53	0.27 - 1.38	210	ATTGCCGATCGCGTGAA-	CCGTTGGCCTTCCTGTAAAG	71
	VIC	*sat-4*	24	1.000 - 1.000	-3.517 - -3.448	0.24 - 1.37	0.38 - 2.10	215	GGCCCAGACCCGTATTCTGT	ACGCCAGCCTGGTCGAT	83
duplex	FAM	*tet(M)*	24	0.999 - 1.000	-3.582 - -3.433	0.18 - 1.73	0.36 - 1.91	525	GAAATCAACCATGTTGATGTAGCATT	CCGGACAATTCAAACAGACCTT	88
	VIC	*tet(O)*	24	0.993 - 0.999	-3.577 - -3.087	0.44 - 1.48	0.34 - 2.08	150	GCTTCCCGGCAACAATTAATAG	CGGCTCCAGATTTATCAGCAAT	98
duplex	Cy5	*16S rRNA gene*	24	0.997 - 0.999	-3.870 - -3.670	0.16 - 0.70	0.58 - 0.70	-	TGGAGAGTTTGATCMTGGCTCAT	CTTTACGCCCARTRAWTCCG	571

Table S2. Relative read abundances of the most abundant genera and species (top 15; [%]) in the tested smoothies. Only read abundances that reached ≥ 0.1% in one of the three technical replicates were considered. "Other" summarizes the read abundances of all remaining genera or species. No sequences retrieved from missing samples.

	**CP**		**F**													
**Genus**	S2	S3	S4	S5	S6	S7	S8	S9	S10	S11	S12	S13	S14	S15	S16	S17
Pseudomonas	2.4	2.6	5.6	2.7	27.8		6.2	8.5	0.2		0.5	16.6	5.3	16.8	1.6	0.5
Tatumella	0.4				0.3				56.2	0.4						
Rahnella	24.6	0.7	1.3						9.5	1.8						
Pantoea	1.4	1.0	0.4	0.2	1.2	0.4	0.8	14.0		0.3	0.7	2.4		1.3		0.1
Glutamicibacter	0.1		9.2		0.2		0.8	0.8			0.8			1.5		
Flavobacterium		0.6	0.7	0.7	0.4		4.3	0.8				0.8	6.3	5.8		0.7
Serratia	7.5	2.9	0.5		2.2		0.8	0.4	1.8	0.4	0.7	0.8		0.1		
Erwinia	2.8	0.2	1.6	0.2	2.6		0.6	0.5	1.2	0.6	0.2	0.9		1.9		
Acinetobacter	0.2	0.4	0.5	0.4	2.7		0.7	0.3			0.3		0.5	4.7		0.3
Duganella	0.3	0.8		1.8			2.4	0.2					1.8	0.8		
Xanthomonas	0.2		0.5					5.0	0.4			0.2		0.4		
Shewanella				0.3	1.4		0.8	0.9						2.9		
Exiguobacterium				0.6	1.9		0.8	0.4						1.3		
Lelliottia	3.4				0.2		0.3		0.7	0.6				0.1		
Other	8.5	1.6	2.3	0.6	1.7	2.9	6.4	3.8	3.8	0.8	1.3	6.1	2.4	1.1	0.5	0.8
**Species**																
Tatumella punctata					0.3				1.3							
Pseudomonas azotoformans		0.6	0.7	0.1	3.9		0.2	0.1			0.5	0.5	0.6	0.3	0.4	1.0
Glutamicibacter arilaitensis	0.6		3.2				0.3	0.2			0.4			0.7		
Xanthomonas citri	0.8		0.2					2.1	0.4			0.6		0.2		
Pantoea vagans	0.2	0.2	0.7	0.6	0.1	0.4	0.2	1.9			0.2	0.4		0.3		0.4
Pseudomonas brassicacearum	0.4		0.1				0.2						0.7	1.4		
Pseudomonas viridiflava	0.3		0.3	1.0			0.9	0.3	0.5			1.1		0.3		
Pseudomonas koreensis	0.2	0.2		0.1	0.3		0.9	0.8				0.1		0.8		
Serratia plymuthica	0.5	0.8	0.1		0.6		0.5	0.2	0.3	0.5	0.1	0.2				
Acinetobacter albensis					0.3			0.4						0.7		
Sphingomonas faeni		0.6	0.4	0.4			0.6				0.9					
Flavobacterium cloacae							0.1							0.5		
Gluconobacter cerinus	0.5		0.4													
Erwinia persicina	0.5		0.9	0.7	0.7		0.2	0.5			0.9	0.3		0.3		
Other	2.3	3.2	1.2	0.7	5.3	0.7	3.5	2.9	1.6	0.5	0.5	2.2	1.9	5.9	0.5	0.8

Table S3. Relative read abundances of the most abundant genera and species (top 15; [%]) in the tested fresh produce. Only read abundances ≥ 0.1% in one of the three technical replicates were considered. "Other" summarizes the read abundances of all remaining genera or species.

	**carrot**				**lettuce**				**tomato**			
**Genus**	O1	O2	C1	C2	O1	O2	C1	C2	O1	O2	C1	C2
Rhodococcus	0.4	0.3	0.8	0.1	0.9	0.5	0.8	0.2				
Massilia					2.2	0.5	0.2	0.1				
Alkanindiges					1.7	0.4						
Xanthomonas					1.5							
Pseudomonas	1.0				0.3	0.3	0.6	0.2				
Stenotrophomonas	0.3	1.0	0.8	0.5	0.2	0.1	0.2					
Pantoea					0.3	0.5	0.9	0.1				
Sphingomonas					0.7	0.8						
Acinetobacter						0.5	0.1	0.6				
Exiguobacterium					0.2	0.3		0.7				
Paracoccus					0.3							
Flavobacterium							0.3					
Allorhizobium					0.3	0.4	0.1					
Rheinheimera							0.2					
Other	1.0		0.6		0.5	0.2	0.2			0.1		0.4
**Species**												
Xanthomonas citri					1.5							
Rhodococcus erythropolis	0.4	0.3	0.8	0.1	0.9	0.5	0.8	0.2				
Sphingomonas faeni					0.5	0.8						
Paracoccus marcusii					0.3							
Pseudomonas koreensis	0.6				0.6		0.3					
Stenotrophomonas rhizophila	0.3	1.0	0.8	0.5	0.1	0.7	0.1					
Pantoea vagans					0.6	0.3		0.1				
Pseudomonas veronii							0.2					
Pantoea agglomerans					0.1	0.2	0.9					
Exiguobacterium acetylicum						0.3						
Massilia oculi							0.2					
Flavobacterium pectinovorum							0.1					
Hymenobacter rigui					0.1							
Acinetobacter johnsonii							0.1					
Other	0.4		0.6		0.5	0.5	0.4	0.3		0.1		0.4

Table S4. Naturally transformable bacteria found in smoothies and fresh produce. Samples were excluded if none of the naturally transformable bacterial genera were detected. Relative read abundances [%] are displayed. Colours represent the highest (red) to lowest (blue) relative read abundances. Genus (1) or species (2) reported in the scientific literature as naturally competent. NA: sequence not assigned.

	Samples	CP		F											lettuce	
		S2	S3	S4	S5	S6	S7	S8	S9	S11	S12	S14	S15	S17	C1	C2
**Genus**	**Species**															
Achromobacter[1]	*xylosoxidans*										0.0002					
	facilis							0.0001				0.0004			0.0035	
Acidovorax[1]	NA		0.0007		0.0004			0.0016	0.0017			0.0035	0.0006			
	valerianellae							0.0007								
Acinetobacter[1]	*calcoaceticus*[2]		0.0025		0.0002	0.0005		0.0002	0.0001							0.0059
	media							0.0002		0.0003			0.0016			
	NA					0.0021				0.0003						
Aeromonas[1]	*rivipollensis*				0.0002	0.0009	0.0001	0.0009	0.0005							
	salmonicida[2]				0.0001	0.0009		0.0020					0.0002			
	sobria					0.0012										
	veronii				0.0001	0.0030		0.0006	0.0003	0.0004			0.0007			
Escherichia	*coli*[2]													0.0010		
Leuconostoc[1]	*carnosum*[2]						0.0001					0.0001				
	lactis													0.0008		
Pseudomonas	*mendocina*[2]		0.0005													
Psychrobacter[1]	*faecalis*															
Streptococcus	*salivarius*[2]								0.0010	0.0007						
	lincolnensis	0.0001											0.0002			
Streptomyces[1]	*microflavus*										0.0008		0.0001			
	scabiei	0.0004														
	turgidiscabies	0.0005		0.0002												

3. Figures:

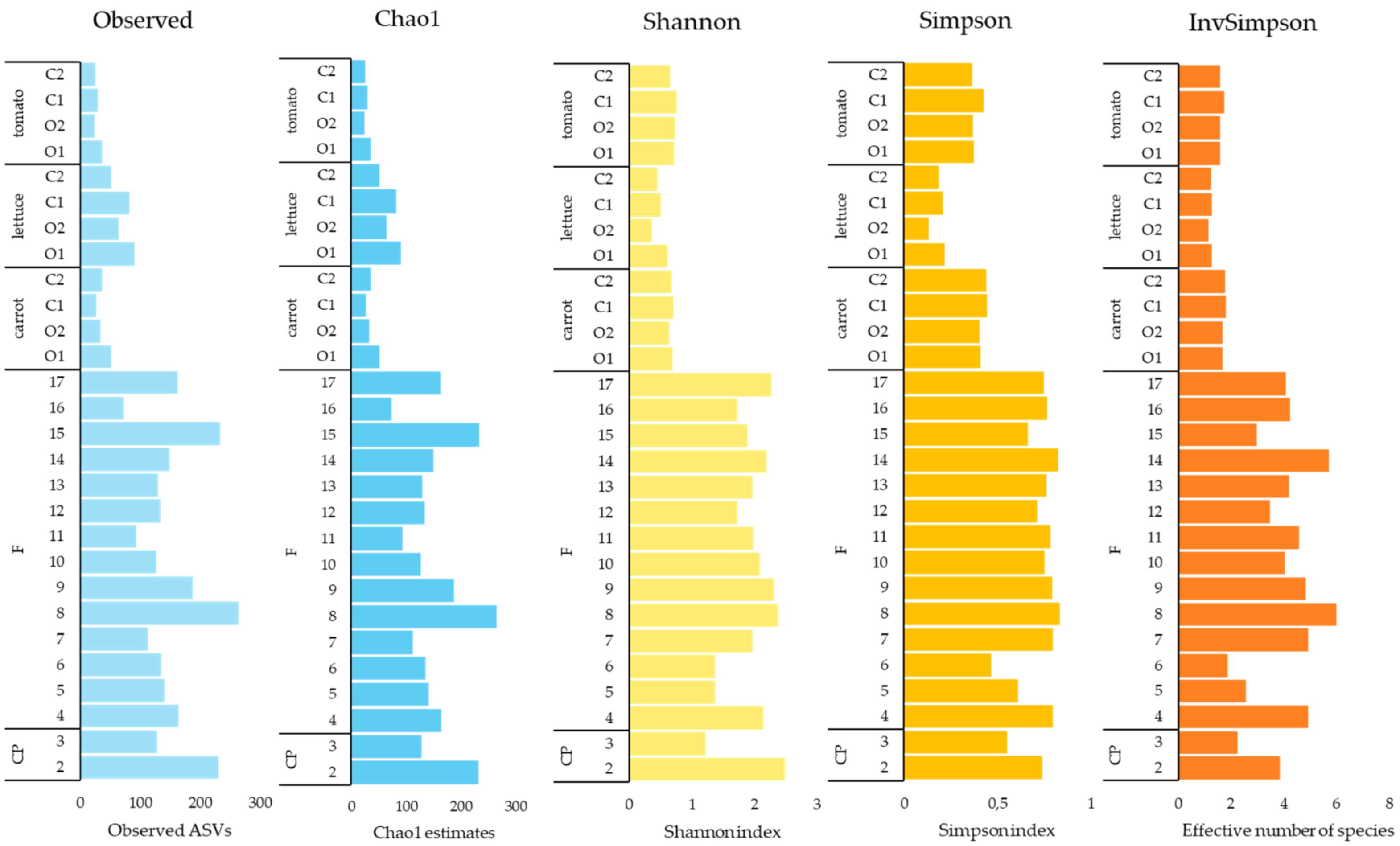

Figure S1. Alpha diversity metrics of bacterial communities in smoothies and fresh produce. C = conventionally grown; CP = cold-pressed; F = freshly-prepared; O = organically fertilized.

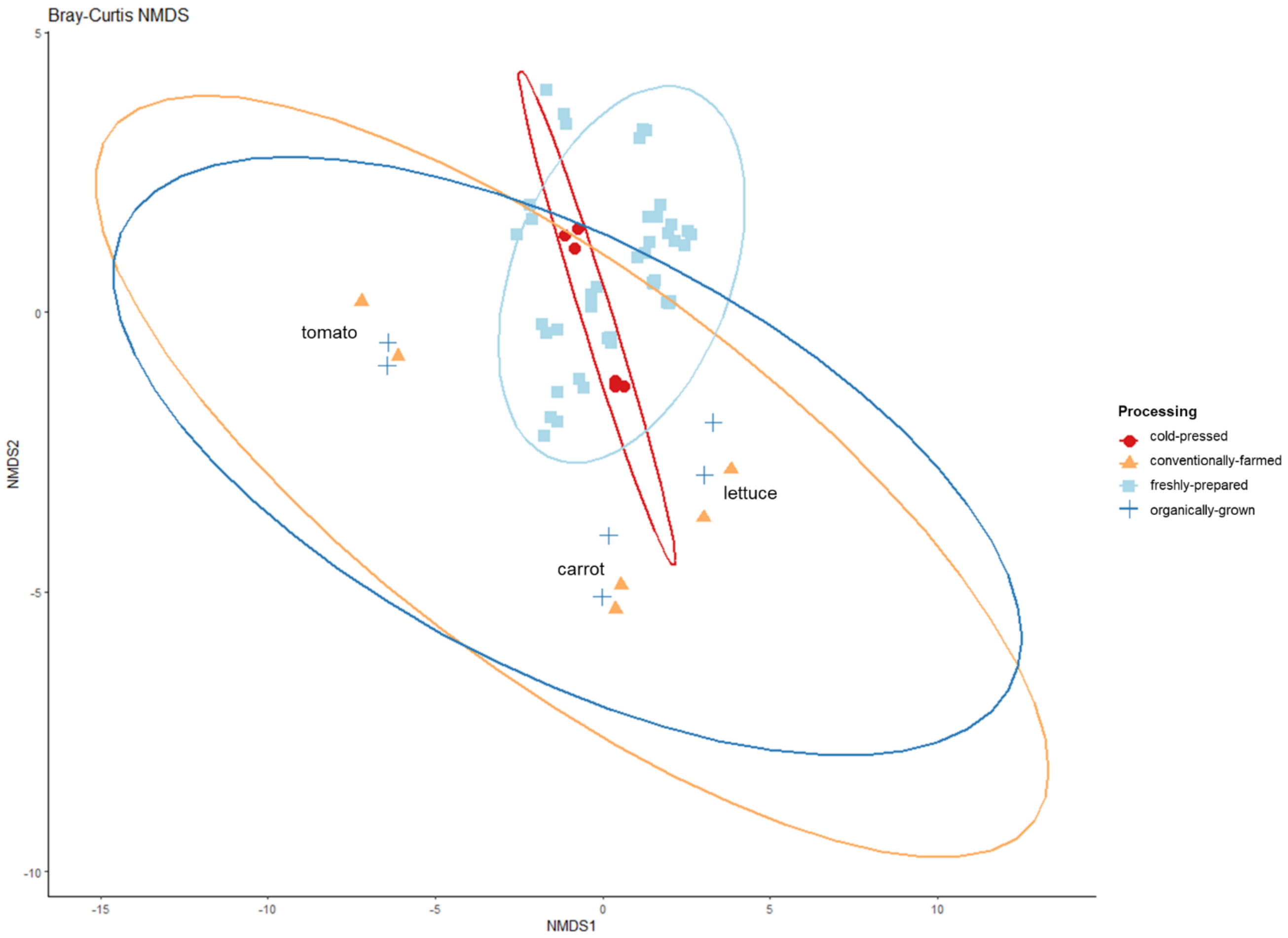

Figure S2. Differences between smoothie and vegetable bacterial communities based on processing status (non-metric multidimensional scaling (NMDS) plots using Bray-Curtis dissimilarity; stress 0.22). Ellipses represent the 95% confidence intervals when comparing the processing status of samples. Cold-pressed and freshly-prepared processing refer to smoothie samples, conventionally-grown and organically cultivated refer to vegetable samples.

			CP		F												
			2	3	4	5	6	7	8	9	10	11	12	13	14	15	17
**Order**	**Genus**	**Species**															
Enterobacterales	*Citrobacter*	*freundii*	0.6								0.9						
		NA	1.8														
	Enterobacter	*hormaechei*	1.0	0.4													
		kobei	0.3														
		NA	1.2	0.2			0.4										
	Escherichia	*coli*															0.7
	Klebsiella	*aerogenes*	0.2								0.3	0.9					
		michiganensis	0.4														
	Serratia	*fonticola*	0.9									0.2					
		liquefaciens					0.6						0.5	0.3			
		NA	4.9								0.5		0.7				
		odorifera														0.1	
		plymuthica	3.2	2.9	0.5		1.6		0.8	0.4	1.3	0.2	0.5	0.5			
Lactobacillales	*Streptococcus*	*salivarius*							0.2								

Figure S3. Relative abundance of potential pathogens in smoothies (with a focus on the WHO bacterial priority pathogens list of 2024 without considering their antibiotic resistance status [38]) as determined by 16S rRNA gene amplicon sequencing. Highlighted cells represent average relative read abundances of ≥ 1%. CP = cold-pressed; F = freshly prepared; NA = sequence not assigned.

Samples	F														CP		P		HPP		carrot				lettuce				tomato			
	S4	S5	S6	S7	S8	S9	S10	S11	S12	S13	S14	S15	S16	S17	S2	S3	S18	S19	S1	S20	O1	O2	C1	C2	O1	O2	C1	C2	O1	O2	C1	C2
**Targets**																																
aadA	-6.9		-6.2			-5.7	-5.9	-5.7		-6.1		-4.9			-0.9						-6.4	-6.8	-7.0	-7.1	-7.1	-6.5	-6.0	-6.1		-6.3		
aph(3′)-IIa						-5.7	-5.4						-4.8																			
aph(3′)-IIIa	-6.4			-5.7			-5.3			-5.6									-0.7		-6.5				-7.9							
$bla_{CTX-M-1-15}$	-5.5	-4.9	-5.8			-4.5		-6.0	-4.3			-4.7	-4.0		-4.2	-3.8																
bla_{KPC}																																
bla_{NDM-1}																																
bla_{OXA-10}																																
bla_{TEM-1}		-6.6	-7.2	-4.5	-5.0		-5.9	-5.6		-6.8	-6.7	-5.8	-6.2	-5.3	-4.3	-5.2	-2.1	-1.9	-1.6	-1.8												
cmxA	-5.2		-5.9		-4.9	-6.1	-5.6	-6.4	-5.9	-5.8	-6.5	-5.1	-5.1	-4.9	-5.2	-5.3					-5.1		-6.8	-7.3	-7.3		-7.0	-7.5				
dfrA-1	-7.2		-7.0					-7.2																				-6.8				
ermB	-6.8		-7.5	-6.0		-6.6	-6.2	-7.2		-6.5	-7.3	-6.7	-5.2		-5.9	-5.2	-4.2	-2.2	-1.3		-6.5	-7.4	-7.5	-7.0	-7.5	-7.5	-7.0	-7.7				
ermF					-4.0	-5.0	-5.8		-5.3										-1.7													
intI1	-5.2	-5.2	-4.7	-5.5	-3.5	-5.0	-4.7	-5.4	-5.0	-5.2	-4.7	-4.7	-4.3	-4.2	-4.1	-4.6	-1.7	-1.3	-0.4	-0.5	-5.8	-6.3	-6.2	-6.6	-6.6	-6.6	-6.1	-5.3		-5.9		
ISPps	-5.1	-4.9	-5.9	-4.7	-3.8	-4.3	-4.0	-5.0	-4.5	-4.4	-4.5	-4.7	-3.9	-3.3	-3.9	-3.7	-0.8	-0.3	0.3	0.6	-5.2	-5.7	-5.4	-5.9	-6.1	-5.7	-5.2	-5.9	-5.0	-5.3	-5.0	-5.6
mcr-1							-6.0						-6.5																			
mecA	-6.5							-6.4																								
qacEΔ1	-6.1	-5.6	-5.3	-6.1	-4.5	-4.4	-5.9	-5.4	-5.2	-5.8	-4.9	-5.0	-4.5	-4.3	-5.3	-5.2	-1.7	-1.5	-1.0		-6.2	-6.8	-6.8	-7.0	-7.6	-6.2	-6.3	-5.7				
qnrS																																
sat-4																																
strB	-6.0		-6.3			-5.2		-5.9		-6.3	-5.6	-5.3	-6.4		-0.2		-5.9			-1.8	-6.7	-6.9	-7.5	-7.8	-7.9	-7.2	-6.5	-6.2				
sul1	-6.0	-6.4	-6.2			-5.8	-5.8	-5.7	-6.0	-5.7	-7.0	-5.0	-5.7	-5.3	-5.6	-5.4		-2.1						-7.5	-7.8	-7.5	-6.5					
tet(A)	-6.7		-6.8									-6.2														-7.5	-7.5					
tet(M)	-6.5	-6.4	-7.3	-6.2		-6.7	-6.0		-6.0	-5.7	-6.2		-5.3			-5.1		-1.9		-1.2	-5.3	-6.7	-6.8	-7.1	-7.3	-7.4	-7.5					
tet(O)	-7.3											-6.8																				
tet(W)	-7.1									-6.7	-7.2	-6.9																				
vanA																																

$2^{-\Delta Ct}$: -8, -7, -6, -5, -4, -3, -2, -1, 0

Smoothie samples

HPP…	high-pressure processed	F…	freshly prepared
CP…	cold-pressed	P…	pasteurized

O …	Organically farmed
C…	Conventionally grown

Figure S4. Relative quantification of ARGs and MGE in smoothie- and fresh produce samples. Ratios of ARG/16S or MGE/16S are calculated using the $2^{-\Delta Ct}$ method and depicted as log10 values.

Relative quantification of ARGs and MGE was performed with the 16S rRNA gene as reference gene (Figure S4). Ratios were obtained using the $2^{-\Delta Ct}$ calculation method [36]. At very low 16S rRNA gene concentrations as occurred in HPP and pasteurized samples, the ratios of detected ARGs and MGE are higher when compared to fresh produce or the freshly prepared and cold-pressed smoothies. The observed low 16S rRNA gene concentrations are most likely caused by the heat- and pressure-based processing methods that induce DNA fragmentation and degradation in these samples [87]. As our 16S rRNA gene amplicon for qPCR has a size of 571 bp, it represents a target-size more sensitive to large-scale fragmentation and degradation of DNA which may lead more frequently to a loss of signal compared to qPCR systems relying on shorter amplicons [87]. Our amplicons for ARGs and MGE are substantially shorter (62 – 137 bp; see Table S1), and, thus, not so sensitive to DNA degradation as induced by HPP and pasteurization.

In summary our 16S rRNA gene quantifications conclusively demonstrate that microbial DNA was drastically fragmented and reduced in pasteurized and HPP samples while shorter ARGs and MGE-amplicon targets were still available for qPCR amplification.

The relative and absolute concentrations obtained from our fresh and cold-pressed smoothies and the fresh produce samples showed comparable results and confirm each other in terms of ARG abundance.

References

36. Schmittgen, T.D.; Livak, K.J. Analyzing Real-Time PCR Data by the Comparative CT Method. *Nat. Protoc.* **2008**, *3*, 1101–1108, doi:10.1038/nprot.2008.73.

38. World Health Organization. WHO Bacterial Priority Pathogens List, 2024: Bacterial Pathogens of Public Health Importance to Guide Research, Development and Strategies to Prevent and Control Antimicrobial Resistance Available online: https://www.who.int/publications/i/item/9789240093461 (accessed on 10 July 2024).

87. Gryson, N. Effect of Food Processing on Plant DNA Degradation and PCR-Based GMO Analysis: A Review. *Anal. Bioanal. Chem.* **2010**, *396*, 2003–2022, doi:10.1007/s00216-009-3343-2.
